# Selection and Validation of Stable Reference Genes for Accurate qRT-PCR Analysis of Flower Color Development in *Rhododendron lapponicum*

**DOI:** 10.3390/cimb48050444

**Published:** 2026-04-24

**Authors:** Liang Xu, Gang Lu, Fangwei Zhou, Congguang Shi, Xiaomei Zhu, Shaozong Yang

**Affiliations:** 1 Zhejiang Key Laboratory of Forest Genetics and Breeding, Zhejiang Academy of Forestry, Hangzhou 310023, China; xuliang@zjforestry.ac.cn (L.X.); zhoufangwei@njfu.edu.cn (F.Z.); shicongguang@zjforestry.ac.cn (C.S.); 2 Zhejiang Forestry—Fund Management Center, Hangzhou 310012, China; 13738037801@163.com; 3 Lin’an District Agriculture and Rural Affairs Bureau, Hangzhou 311300, China; zxm15906641535@163.com

**Keywords:** *Rhododendron lapponicum*, quantitative real-time PCR, reference gene, flower color, anthocyanin biosynthesis, gene expression normalization

## Abstract

*Rhododendron lapponicum* (L.) Wahlenb., prized for its vibrant and diverse floral displays, holds significant ornamental and ecological value. However, advances in its molecular breeding have been constrained by the absence of reliable tools for accurate gene expression analysis. A fundamental requirement for such studies is the identification of stable reference genes for qRT-PCR. To date, no systematically validated reference genes exist for normalizing gene expression across *R. lapponicum* cultivars with diverse flower colors, representing a major technical obstacle to elucidating the molecular mechanisms of color formation. This study aimed to fill this gap by systematically identifying and validating optimal reference genes for petal tissues in six distinct *R. lapponicum* cultivars. We assessed the expression stability of 11 candidate genes using four independent algorithms and integrated the results via RefFinder. Our comprehensive analysis across multiple algorithms consistently identified *RlaEF1-α* and *RlaACT* as the most stably expressed reference genes. Their reliability was robustly validated by normalizing the expression of *RlaMYB113*, a key anthocyanin regulator; the normalized expression levels showed an extremely significant difference between rose-red and white cultivars (*p* < 0.001) and produced a coherent, phenotype-correlated profile, in contrast to the distorted patterns obtained with unstable references. This study establishes *RlaEF1-α* and *RlaACT* as a precise dual-gene internal control for qRT-PCR. By providing a validated normalization framework, our work enables accurate quantification of color-related genes and directly supports molecular breeding efforts aimed at the targeted development and selection of novel *R. lapponicum* cultivars with desirable and stable flower colors.

## 1. Introduction

*Rhododendron lapponicum* (L.) Wahlenb., a perennial evergreen dwarf shrub within the genus *Rhododendron* (Ericaceae), is renowned for its early spring blossoms, diverse floral morphology, and vibrant colors [1]. It plays a significant role in urban landscaping, potted ornamental use, and alpine ecosystem restoration [2]. With accelerating urbanization and growing demands for ecological enhancement, the market for ornamental plants—particularly novel cultivars with stable and rich color variations—is expanding rapidly. However, current breeding programs for *R. lapponicum* still predominantly rely on conventional hybridization and phenotypic selection, which are hampered by lengthy breeding cycles and unstable inheritance of floral color traits [3]. Therefore, elucidating the biological mechanisms underlying color formation and employing molecular-assisted breeding strategies have become urgent avenues for developing new cultivars with stable, desirable traits and independent intellectual property. This approach holds clear practical significance for enhancing the competitiveness of China’s ornamental horticulture industry and improving the quality of ecological landscapes.

Floral color, one of the most critical visual traits in plants, is orchestrated by a sophisticated, multi-layered regulatory network involving the synthesis, modification, and transport of anthocyanins, as well as the pH of the vacuolar microenvironment [4]. Elucidating these regulatory mechanisms is a fundamental prerequisite for the targeted modification of floral coloration. In recent years, quantitative real-time polymerase chain reaction (qRT-PCR) has emerged as a cornerstone technique for deciphering gene expression patterns and mapping regulatory networks, owing to its high sensitivity, specificity, and accurate quantification capability [5]. The reliability of this technique fundamentally depends on the use of reference genes that exhibit stable expression across varied experimental conditions, which are essential for normalizing variations in RNA yield, quality, and reverse transcription efficiency [6]. Fluctuations in the expression of these reference genes can lead to the failure of proper data normalization for target genes, consequently yielding misleading conclusions. Currently, molecular biological research on *R. lapponicum* remains in its nascent stages, and there is a notable lack of a systematically screened and validated set of reference genes tailored to its specific biological contexts, such as floral color variation.

Although genes such as Actin, UBQ (ubiquitin), and GAPDH (glyceraldehyde-3-phosphate dehydrogenase) are commonly used as reference genes in other ornamental plants like Arabidopsis and rose [7,8], their direct application in studies of *R. lapponicum* carries significant risks. First, the genome of *R. lapponicum* exhibits polyploidy and contains abundant repetitive sequences, which may lead to divergent expression patterns among members of the same gene family [9]. Second, floral color formation involves a specific and dynamically regulated metabolic and transcriptional network, whose strong activity could disturb the stability of conventional “housekeeping” genes [10]. More critically, genetic background and physiological status likely differ among cultivars with distinct flower colors, further complicating the identification of reference genes that perform consistently across cultivars, tissues, and developmental stages [11]. Therefore, adopting reference genes from other species may fail to accurately reflect the true expression dynamics of color-related genes in *R. lapponicum*, which would severely limit in-depth investigation of its floral color molecular mechanisms and their application in breeding.

The systematic screening and validation of a set of reference genes suitable for gene expression analysis across different flower-color cultivars of *R. lapponicum* represents a critical and fundamental task to overcome current technical limitations. This study aims to identify the optimal reference gene or combinations thereof by evaluating the expression stability of multiple candidate reference genes in *R. lapponicum* cultivars exhibiting diverse floral colors. This work will not only provide a reliable technical foundation for the precise dissection of the floral color regulatory network in this species, significantly enhancing the accuracy and comparability of qRT-PCR data, but will also establish a methodological basis for molecular studies on other important traits (e.g., stress resistance, flowering period) in *R. lapponicum*. Ultimately, this research will strongly facilitate the transition of *R. lapponicum* breeding from traditional empirical approaches to molecular design-based strategies, accelerating the development of novel cultivars that meet diverse market demands. It holds substantial scientific value and application prospects for promoting the sustainable development and enhancing the international competitiveness of the *R. lapponicum* industry.

## 2. Materials and Methods

### 2.1. Plant Materials

**Figure 1:**
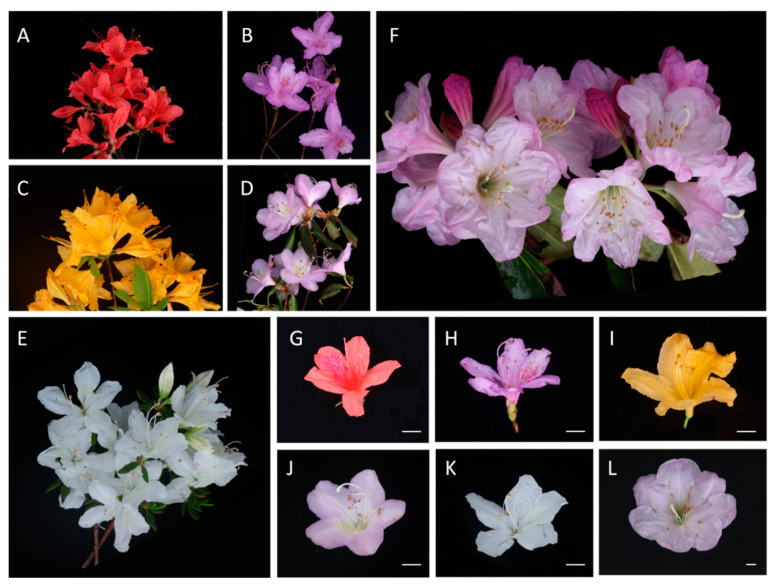
Flower samples of six *Rhododendron* cultivars with distinct flower colors. (**A**,**G**) Inflorescence and individual flower of *R. simsii*. (**B**,**H**) Inflorescence and individual flower of *R. mariesii*. (**C**,**I**) Inflorescence and individual flower of *R. latoucheae*. (**D**,**J**) Inflorescence and individual flower of *R. molle*. (**E**,**K**) Inflorescence and individual flower of *R. mucronatum*. (**F**,**L**) Inflorescence and individual flower of *R. fortunei*. Scale bar: 1 cm.

In April 2024 (full-bloom stage), petal samples were collected from six distinct cultivars of *R. lapponicum* Wahlenb. exhibiting different flower colors, as shown in Figure 1 (A–F for inflorescences, G–L for individual flowers). Their flower colors included rose red, yellow, varying shades of light pink, and white. All plants were grown under identical conditions, including the same soil type, natural light exposure, and ambient temperature. To ensure the independence of biological replicates, at least three individual plants (*n* = 3) with similar growth conditions were sampled per species, with each plant considered as one biological replicate. Immediately after collection, petal samples were placed in pre-labeled sterile cryotubes and flash-frozen in liquid nitrogen in the field. All samples were subsequently transported to the laboratory and stored long-term at −80 °C in an ultra-low temperature freezer until total RNA extraction.

### 2.2. Total RNA Extraction and cDNA Synthesis

Total RNA was isolated from petal samples of the six aforementioned *Rhododendron* cultivars using the RNAprep Pure Polysaccharide Polyphenol Plant Total RNA Extraction Kit (TIANGEN, Nanjing, China). The concentration and purity (A260/A280 ratio > 2.0) of the extracted RNA were subsequently assessed with a NanoDrop 1000 spectrophotometer (Thermo Fisher Scientific, Waltham, MA, USA) using the operating software version 3.8.1. RNA integrity was verified by 1.0% agarose gel electrophoresis, confirming clear bands with no evident degradation. Complementary DNA (cDNA) for subsequent qRT-PCR analysis was synthesized via reverse transcription. The procedure followed the instructions of the One-Step gDNA Removal and cDNA Synthesis SuperMix kit (TransGen, Nanjing, China). Specifically, 1 μg of total RNA was used as template in a 20 μL reaction system to simultaneously remove genomic DNA and synthesize first-strand cDNA. The reaction program was strictly set according to the manufacturer’s protocol. The resulting cDNA products were stored at −20 °C for subsequent use.

### 2.3. Selection of Candidate Reference Genes and Primer Design

To establish a reliable reference gene system for *R. lapponicum*, 11 candidate genes were selected based on two principles: functional diversity and conserved stability. The functional categories covered include cytoskeletal (*ACT*, *α-TUB1*, *α-TUB2*), translational (*EF1-α*), protein degradation (*OTU*), chromatin-related (*H2A*, *HIS*), signaling (*PP2A*), cell cycle (*CDC2*), ATPase (*VHAC*), and chaperone (*DnaJ*) genes. The conserved stability principle ensures that all selected genes have been widely used as reference genes in qRT-PCR studies across various plant species, including *Rhododendron* and other ornamentals [12,13,14], thereby supporting their potential applicability in *R. lapponicum*. This combined strategy minimizes the risk of co-regulation bias and increases the likelihood of identifying stably expressed internal controls.

To identify the precise homologs in *R. lapponicum*, BLASTn searches (version 2.12.0+) were performed against the published *R. lapponicum* transcriptome assembly (NCBI BioProject PRJNA594084) [2] with the following parameters: e-value ≤ 1 × 10^−5^, percent identity ≥ 70%, and query coverage ≥ 70%. For each candidate gene family, the best hit (i.e., the highest bit score and lowest e-value) was selected, and its full-length transcript sequence was retrieved. Gene-specific primers were designed using Primer Premier software (version 5.0, Premier Biosoft, Palo Alto, CA, USA). All primers were commercially synthesized by Sangon Biotech Co., Ltd. (Shanghai, China). Primer validation was conducted in a two-step process. First, conventional PCR amplification was performed using pooled cDNA templates. The resulting amplicons were analyzed by agarose gel electrophoresis and visualized with a gel imaging system to confirm the presence of a single band of the expected size and the absence of primer-dimers or non-specific products. Second, the specificity of the primers was further rigorously assessed via qRT-PCR by analyzing the melt curve profiles. Only primer pairs that generated a single, sharp dissociation peak, with no signal detected in negative control (no-template) reactions, were selected for use in subsequent gene expression analyses.

### 2.4. Construction of Standard Curves and Evaluation of Primer Amplification Efficiency

To evaluate the amplification efficiency of each candidate reference gene primer pair and to establish a basis for subsequent relative quantification, a standard curve was constructed for each primer set. A pooled cDNA template, prepared by combining equal amounts of cDNA from all samples, was subjected to a five-fold serial dilution, generating five concentration gradients (undiluted, 1:5, 1:25, 1:125, and 1:625). Each dilution was analyzed in triplicate (technical replicates), alongside a negative control containing ddH_2_O instead of template, to monitor potential contamination. qRT-PCR was performed using a QIAquant 96 2plex Real-Time PCR System (QIAGEN, Hilden, Germany) with the QIAquant 96 Software (version 1.0.3). Standard curves were generated by plotting the quantification cycle (Cq) values against the logarithm of the corresponding template dilution factor. Linear regression analysis was performed using the instrument’s accompanying software to obtain the slope of each standard curve. The amplification efficiency (E) for each primer pair was calculated according to the formula: E = [10^(−1/slope) − 1] × 100%. Primer pairs exhibiting an amplification efficiency between 90% and 110%, coupled with a standard curve correlation coefficient (R^2^) greater than 0.98, were considered technically suitable for reliable quantitative analysis and were subsequently used for expression stability evaluation [15].

### 2.5. Quantitative Real-Time PCR Analysis

qRT-PCR was conducted on a QIAquant 96 2plex Real-Time PCR System (QIAGEN, Germany). The reaction mixture was prepared in a final volume of 20 μL, consisting of 10 μL of PowerUp™ SYBR™ Green Master Mix (Thermo Fisher Scientific, USA), forward and reverse primers at a final concentration of 4 nmol/L each, 2 μL of cDNA template (equivalent to 50 ng of total RNA-derived cDNA), and nuclease-free ddH_2_O to make up the remaining volume. All reactions were performed in triplicate as technical replicates, and each run included a no-template control (NTC) to assess potential contamination. The thermal cycling conditions were as follows: an initial denaturation at 95 °C for 3 min, followed by 40 cycles of denaturation at 95 °C for 15 s, annealing at 60 °C for 15 s, and extension at 72 °C for 30 s. To verify amplification specificity, a melt curve analysis was performed immediately after the PCR by heating the products from 55 °C to 95 °C with a continuous fluorescence measurement at a ramp rate of 0.3 °C/s.

### 2.6. Data Processing and Stability Analysis of Candidate Reference Genes

To comprehensively and reliably evaluate the expression stability of the candidate reference genes, four widely used algorithms were employed in combination, and a comprehensive ranking was generated via the RefFinder online platform.

(1) ΔCt Method Analysis: This method evaluates stability by comparing the variation in ΔCt values (i.e., the difference in quantification cycle, Cq, between any two genes) across different samples. Constant ΔCt values for a gene pair across samples indicate stable co-expression of both genes. By systematically comparing the variability of ΔCt values among all candidate gene pairs, a stability ranking for all genes can be derived [16].

(2) BestKeeper Analysis: This algorithm (version 1.00) assesses stability based on pairwise correlation and regression analyses, as described in Pfaffl et al. (2004) [17]. It calculates the standard deviation (SD) and coefficient of variation (CV) of the Cq values for each candidate gene. Lower SD and CV values indicate smaller fluctuations in expression across samples and, consequently, higher stability [17].

(3) NormFinder Analysis: This method employs a model-based variance estimation approach to calculate a stability value for each candidate gene. This value directly reflects the intra-group and inter-group variation in gene expression. The analysis was performed using NormFinder.xla (version 0.953). The gene with the lowest stability value is considered the most stable reference gene [18].

(4) geNorm Analysis: This algorithm (as described in Vandesompele et al., 2002 [19]) utilizes a pairwise comparison strategy to calculate an expression stability measure (M value) for each gene. The analysis was performed using the geNorm algorithm module within the qbasePLUS software (version 3.0, Biogazelle, Ghent, Belgium). A lower M value indicates greater expression stability, with M < 1.5 commonly accepted as the threshold for reliable stability [19]. Furthermore, geNorm determines the optimal number of reference genes by calculating the pairwise variation (Vn/n + 1) between consecutive normalization factors. A Vn/n + 1 ratio < 0.15 suggests that including the (n + 1)th gene does not significantly improve normalization accuracy, indicating that the first n genes constitute the optimal combination. If the ratio is ≥0.15, the inclusion of the (n + 1)th gene is recommended [20].

(5) Comprehensive Ranking: To obtain the most robust evaluation, the results from the four algorithms above were integrated using the online tool RefFinder (https://www.heartcure.com.au/reffinder/, accessed on 16 September 2024) [21]. RefFinder assigns a weight to the ranking of each gene derived from each algorithm, calculates their geometric mean, and ultimately produces a comprehensive ranking. This final ranking is used to recommend the optimal reference gene or gene combination. A schematic overview of the complete analytical pipeline is presented in Figure S1.

### 2.7. Validation of Reference Gene Stability

To validate the effectiveness of the selected reference genes in a practical research context, particularly for gene expression analysis related to flower color formation, a verification experiment was designed. The target gene *RlaMYB113*, a putative MYB transcription factor involved in anthocyanin biosynthesis, was identified from the *R. lapponicum* transcriptome assembly (NCBI BioProject PRJNA594084) via homology-based BLASTn searches using *Arabidopsis thaliana MYB113* as the query. This gene was chosen as a key regulator in the anthocyanin biosynthesis pathway contributing to flower pigmentation [22]. The relative expression level of *RlaMYB113* in petals from the six *Rhododendron* cultivars with distinct flower colors was calculated using two different normalization strategies: the most stable reference gene combination identified in this study (*RlaEF1-α* and *RlaACT*), and a combination of less stable reference genes (*RlaPP2A* and *RlaHIS*). By comparing the resulting expression profiles of *RlaMYB113* normalized with these different references, the impact of reference gene stability on target gene expression analysis was directly assessed. This comparative approach validates the reliability and practical advantage of the optimal reference gene combination recommended by this study for molecular investigations into flower color mechanisms.

## 3. Results

### 3.1. RNA Quality Testing and Primer Specificity Validation

High-quality total RNA was successfully isolated from petals of six Rhododendron cultivars exhibiting distinct flower colors (Figure 1). The RNA concentrations ranged from 140 to 400 ng/μL, with OD260/280 ratios between 2.0 and 2.3, confirming high purity without contamination from proteins or phenolic compounds. Electrophoretic analysis on a 1.0% agarose gel revealed intact ribosomal RNA bands, with a clear 28S:18S brightness ratio approximating 2:1, indicating minimal degradation and high integrity of the RNA samples. These results confirm that the extracted RNA was suitable for subsequent cDNA synthesis and gene expression analysis. A total of 11 candidate reference genes were selected based on homology to conserved housekeeping genes reported in related plant species. Gene-specific primers were designed from full-length transcript sequences of Rhododendron and validated for specificity using BLASTn searches (version 2.12.0+) analysis. Initial screening by conventional PCR followed by agarose gel electrophoresis demonstrated that all primers produced single amplicons of expected sizes (126–222 bp) without primer-dimer or non-specific amplification (Figure S2). Further validation by qRT-PCR showed single-peak dissociation curves for each candidate gene, confirming amplification specificity and absence of secondary products (Figure S3). Consistent amplification curves across technical replicates indicated optimal annealing temperatures and robust primer performance (Figure S4).

The amplification efficiency (E) and correlation coefficients (R^2^) for each primer pair were determined using serial dilutions of pooled cDNA templates. As summarized in Table 1, the amplification efficiencies ranged from 97.43% to 118.27%, with most falling within the acceptable range of 90–110%. *RlaPP2A* (116.61%) and *RlaHIS* (118.27%) exhibited amplification efficiencies slightly above the recommended 110% threshold, suggesting potential non-optimal primer performance or secondary structure artifacts. The linear correlation coefficients (R^2^) varied between 0.9738 and 0.9953, indicating strong linearity across the dilution series. These results confirm that all designed primers exhibit high specificity, consistent amplification efficiency, and reliable performance in qRT-PCR assays, providing a solid foundation for subsequent stability evaluation of reference genes in Rhododendron. In addition to reference genes, primers for the target gene *RlaMYB113*, a putative MYB transcription factor implicated in flower pigmentation, were also designed and validated. The primers demonstrated an amplification efficiency of 100.37% and an R^2^ value of 0.9921, with a single specific product of 211 bp (Table 1). These results confirm the suitability of the *RlaMYB113* primer set for accurate expression profiling in subsequent experiments. Overall, the systematic validation of RNA quality, primer specificity, amplification efficiency, and linear dynamic range ensures the reliability of the qRT-PCR platform for studying gene expression in Rhododendron, particularly for the selection of stable reference genes under varied experimental conditions.

### 3.2. Reference Primer Amplification Ct Value Analysis

**Figure 2:**
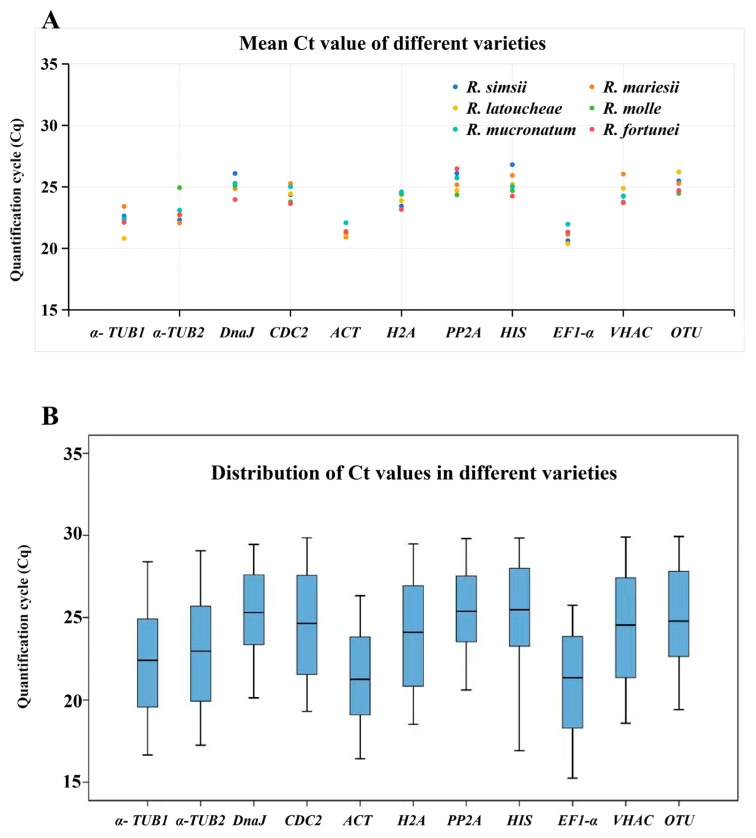
Distribution of Ct values for candidate reference genes across *Rhododendron* petal samples. (**A**) Mean Ct values of the 11 candidate reference genes calculated from different Rhododendron cultivars. (**B**) Box plots depicting the Ct value distribution for each candidate gene. The horizontal line inside the box represents the median, the box spans the interquartile range, and the whiskers extend to the minimum and maximum Ct values.

The cycle threshold (Ct) values obtained from qRT-PCR analysis provide a direct measure of initial transcript abundance, with lower Ct values indicating higher expression levels. Analysis across all experimental samples (six *Rhododendron* cultivars with distinct flower colors) revealed a broad distribution of Ct values for the 11 candidate reference genes, with an overall range from 15.25 to 29.94 (Table S1). This span confirms that the selected genes cover a wide spectrum of expression abundances, a necessary condition for identifying robust normalizers under varying experimental contexts. Distinct expression patterns were observed among the candidate genes (Figure 2). *RlaEF1-α* consistently showed the highest expression, with Ct values across cultivars ranging from 15.25 to 25.73. *RlaACT* also exhibited high expression, with Ct values between 16.42 and 26.33. In contrast, *RlaPP2A* and *RlaHIS* displayed the lowest expression levels, with Ct values as high as 29.80 and 29.84, respectively, in certain samples. For example, in the *R. simsii* series, the average Ct for *RlaEF1-α* was 20.63, whereas for *RlaHIS* it was 26.81, clearly illustrating the abundance difference. For instance, *RlaEF1-α* showed relatively consistent expression across most samples, while genes such as *RlaPP2A* exhibited wider variation, suggesting higher sensitivity to experimental conditions or variety differences. Notably, within individual cultivars, certain genes demonstrated notable expression shifts across treatment levels. In *R. molle*, *RlaHIS* varied from 16.91 to 28.61, indicating potentially treatment-responsive expression, which may disqualify it as a stable reference. In summary, the Ct value analysis established the expression landscape of the candidate reference genes, revealing significant differences in both abundance and apparent stability across samples and treatments. These quantitative findings underscore that expression stability cannot be inferred from mean Ct value alone and necessitate a more rigorous, algorithm-based stability evaluation to definitively identify the optimal reference genes for reliable normalization in subsequent gene expression studies on *Rhododendron*.

### 3.3. Analysis of Gene Expression Stability

We systematically evaluated the expression stability of 11 candidate reference genes across six *Rhododendron* cultivars with varying flower colors. To obtain a robust and comprehensive stability ranking, multiple algorithm-based approaches were employed, including the comparative ΔCt method, BestKeeper, NormFinder, and geNorm. These methods assess stability based on different statistical principles. This multi-algorithm strategy enhances the credibility of the selected reference genes, ensuring their suitability for normalizing target gene expression in subsequent studies on flower color development and related molecular mechanisms in *Rhododendron*.

#### 3.3.1. Stability Ranking of Candidate Reference Genes via the Delta Ct Method

To systematically evaluate the expression stability of the candidate reference genes across six distinct Rhododendron cultivars, the comparative ΔCt method was employed. This approach assesses stability based on the pairwise variation in Ct values between genes within each sample; a lower average standard deviation of ΔCt (denoted as SDΔCt) indicates higher expression stability. The stability ranking derived from the ΔCt analysis is presented in Table 2. Among the 11 candidates, *RlaACT* exhibited the highest stability with SDΔCt = 0.59, followed closely by *RlaEF1-α* (SDΔCt = 0.63). These two genes demonstrated minimal variation in expression across all tested conditions and cultivars. *Rlaα-TUB1* (SDΔCt = 0.72) and *RlaOTU* (SDΔCt = 0.74) also showed relatively stable expression profiles. In contrast, genes such as *RlaPP2A* (SDΔCt = 1.41) and *RlaHIS* (SDΔCt = 1.55) displayed considerably higher variability, marking them as the least stable under the experimental settings. The overall stability order was determined as follows: *RlaACT* > *RlaEF1-α* > *Rlaα-TUB1* > *RlaOTU* > *RlaVHAC* > *RlaDnaJ* > *RlaCDC2* > *Rlaα-TUB2* > *RlaH2A* > *RlaPP2A* > *RlaHIS*. The analysis results of ΔCt indicate that *RlaACT* and *RlaEF1-α* are the most consistently expressed genes across different Rhododendron cultivars and are therefore recommended as optimal reference genes for normalizing target gene expression in subsequent qRT-PCR studies related to flower color development in this genus.

#### 3.3.2. Stability Evaluation of Candidate Reference Genes via BestKeeper Analysis

To further assess the expression stability of candidate reference genes, the BestKeeper algorithm was employed. A gene is generally considered stably expressed when its SD is below the threshold of 1.0. As detailed in Table 2, among the 11 candidate genes, six exhibited SD values below 1.0, meeting the criterion for stable expression. *RlaEF1-α* demonstrated the highest stability with the lowest SD (0.64) and CV (2.71). It was closely followed by *RlaACT* (SD = 0.72, CV = 2.94). *RlaCDC2*, *RlaDnaJ*, *Rlaα-TUB1*, and *RlaH2A* also showed favorable stability, with SD values ranging from 0.78 to 0.98. Conversely, the remaining five genes displayed SD values exceeding 1.0, indicating substantial expression variability. *Rlaα-TUB2*, *RlaVHAC*, and *RlaOTU* had moderate variability (SD: 1.17–1.41), while *RlaPP2A* (SD = 1.78) and *RlaHIS* (SD = 2.11) were identified as the most unstable genes, with *RlaHIS* also showing the highest CV (6.85). Based on the SD and CV values, the overall stability ranking determined by BestKeeper was: *RlaEF1-α* > *RlaACT* > *RlaCDC2* > *RlaDnaJ* > *Rlaα-TUB1* > *RlaH2A* > *Rlaα-TUB2* > *RlaVHAC* > *RlaOTU* > *RlaPP2A* > *RlaHIS*. This result strongly supports *RlaEF1-α* and *RlaACT* as the most reliable reference genes, corroborating the findings from the ΔCt method, while highlighting *RlaHIS* and *RlaPP2A* as unsuitable for normalization under the tested experimental conditions.

#### 3.3.3. Expression Stability Analysis Using NormFinder

The expression stability of the candidate reference genes was further evaluated using the NormFinder algorithm, which calculates a stability value (S-value) for each gene by considering both intra- and inter-group variations. A lower S-value indicates higher expression stability. As summarized in Table 2, *RlaACT* exhibited the highest stability with the lowest S-value (0.109), followed closely by *RlaEF1-α* (S-value = 0.117). These results reinforce their suitability as robust internal controls. *Rlaα-TUB1* (0.207), *RlaCDC2* (0.214), and *RlaDnaJ* (0.306) also demonstrated relatively stable expression profiles. In contrast, genes such as *RlaH2A* (0.669), *RlaOTU* (0.718), and *RlaPP2A* (0.817) showed moderate to high variability. Consistent with previous analyses, *RlaHIS* was identified as the least stable gene, with the highest S-value of 0.982. The stability ranking derived from NormFinder was as follows: *RlaACT* > *RlaEF1-α* > *Rlaα-TUB1* > *RlaCDC2* > *RlaDnaJ* > *RlaVHAC* > *Rlaα-TUB2* > *RlaH2A* > *RlaOTU* > *RlaPP2A* > *RlaHIS*. The strong concordance between NormFinder and the previously applied ΔCt and BestKeeper analyses—particularly in identifying *RlaACT* and *RlaEF1-α* as the top candidates—further validates their exceptional expression consistency across different Rhododendron cultivars and experimental conditions, thereby supporting their selection as optimal reference genes for subsequent gene expression normalization.

#### 3.3.4. Determination of Optimal Reference Gene Number and Stability Using geNorm Analysis

**Figure 3:**
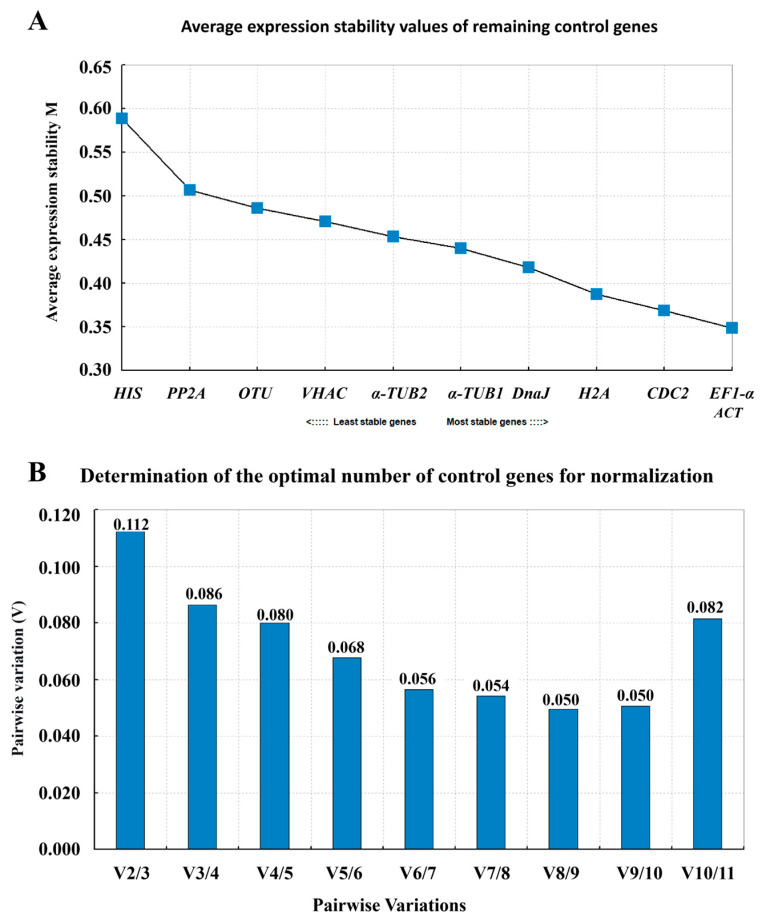
Evaluation of reference gene stability using the geNorm algorithm. (**A**) Expression stability analysis of 11 candidate reference genes. The average expression stability value (M) was calculated by geNorm, where lower M values indicate higher expression stability. Genes are ranked from the most stable (left) to the least stable (right). (**B**) Determination of the optimal number of reference genes for accurate normalization. Pairwise variation analysis (Vn/n + 1) was performed, with a cut-off value set at 0.15 (dashed line). The point where Vn/n + 1 falls below 0.15 indicates the recommended number of reference genes for reliable normalization.

To further validate the stability of candidate reference genes and determine the optimal number required for reliable normalization, the geNorm algorithm was applied. This method calculates the average expression stability measure (M), with lower M values indicating higher stability. An M value below 1.5 is generally considered acceptable for a reference gene. As shown in Figure 3A, Table S2, all 11 candidate genes exhibited M values well below the threshold of 1.5, confirming their overall stability under the experimental conditions. *RlaEF1-α* and *RlaACT* demonstrated the lowest M values, indicating they are the most stably expressed genes across all tested Rhododendron cultivars. This result aligns consistently with findings from the ΔCt, BestKeeper, and NormFinder analyses, reinforcing the robustness of these two genes as superior internal controls.

To determine the optimal number of reference genes for normalization, geNorm calculates the pairwise variation Vn/n + 1) between sequential normalization factors. A value below 0.15 suggests that the inclusion of an additional reference gene is unnecessary. In this study, the V2/3 value was calculated to be 0.112, which is below the recommended cutoff of 0.15 (Figure 3B, Table S2). This indicates that the use of two reference genes is sufficient to achieve stable and accurate normalization for qRT-PCR analysis across different Rhododendron flower color variants.

#### 3.3.5. Comprehensive Stability Ranking of Candidate Reference Genes via RefFinder Analysis

Given that different stability assessment algorithms employ distinct statistical principles and may yield varying rankings, we utilized the RefFinder web tool to integrate the results from ΔCt, BestKeeper, NormFinder, and geNorm analyses. RefFinder calculates a comprehensive stability ranking based on the geometric mean of the rankings from all four methods, providing a more robust and authoritative evaluation. The consolidated ranking, derived from the geometric mean values, is presented in Table 3. *RlaACT* and *RlaEF1-α* emerged as the two most stable genes, securing the first and second positions in the overall ranking, respectively. This conclusion is strongly supported by their consistent top-tier performance across all four individual algorithms. Following these top candidates, *Rlaα-TUB1*, *RlaCDC2*, and *RlaDnaJ* ranked third to fifth, showing relatively stable expression profiles. Conversely, *RlaPP2A* and *RlaHIS* were consistently identified as the least stable genes, occupying the final two positions in the comprehensive ranking. The overall stability order determined by RefFinder was as follows: *RlaACT* > *RlaEF1-α* > *Rlaα-TUB1* > *RlaCDC2* > *RlaDnaJ* > *RlaVHAC* > *Rlaα-TUB2* > *RlaH2A* > *RlaOTU* > *RlaPP2A* > *RlaHIS*. This integrative analysis reinforces that *RlaACT* and *RlaEF1-α* represent the optimal reference gene combination for normalizing qRT-PCR data across diverse Rhododendron cultivars. Their exceptional and consistent stability minimizes potential normalization errors, thereby ensuring the accuracy and reliability of subsequent studies on gene expression related to flower color development in this genus.

### 3.4. Validation of Selected Reference Genes Using a Target Gene Involved in Anthocyanin Biosynthesis

**Figure 4:**
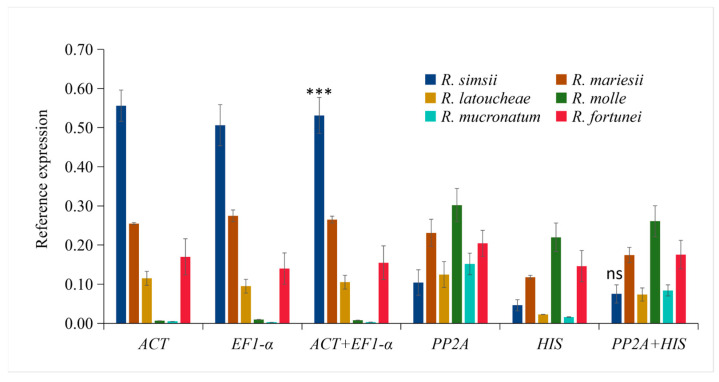
Validation of reference gene stability using *RlaMYB113* expression in *Rhododendron* petals. Relative expression levels (mean ± SEM, n = 3 biological replicates) were normalized with the stable combination (*RlaEF1-α* + *RlaACT*) or the unstable combination (*RlaPP2A* + *RlaHIS*). *** *p* < 0.001 (two-tailed *t*-test, rose-red vs. white); ns, not significant (*p* = 0.42).

To experimentally validate the reliability of the identified optimal reference genes, we analyzed the expression pattern of *RlaMYB113*, a key MYB transcription factor regulating anthocyanin biosynthesis, across six Rhododendron cultivars with distinct flower colors. The relative expression of *RlaMYB113* was normalized using two different sets of controls: the combination of the top-ranked stable genes, *RlaEF1-α* and *RlaACT*; and the unstable genes, *RlaPP2A* and *RlaHIS*, for comparison. When normalized against the stable *RlaEF1-α* and *RlaACT*, the expression profile of *RlaMYB113* exhibited a clear and biologically consistent pattern (Figure 4). The highest transcript levels were detected in the rose-red cultivar (*R. simsii*), with progressively lower levels in pink (*R. mariesii*, *R. molle*, *R. fortunei*) and yellow cultivars (*R. latoucheae*), and the lowest expression in the white cultivar (*R. mucronatum*). Statistical analysis using a two-tailed independent *t*-test (Welch’s correction) on three biological replicates revealed that the expression level in the rose-red cultivar was significantly higher than that in the white cultivar (*p* < 0.001). In contrast, normalization using the unstable reference genes RlaPP2A and RlaHIS yielded a markedly different and inconsistent expression profile (Figure 4). The resulting data showed high variability and lacked a coherent trend correlating with flower color phenotype. The same comparison between rose-red and white cultivars using the unstable normalizers showed no significant difference (*p* = 0.42). These results demonstrate that the use of unstable reference genes can distort the apparent expression pattern of a target gene, potentially leading to erroneous biological interpretations. The clear, logical, and phenotype-correlated expression pattern obtained with *RlaEF1-α* and *RlaACT* confirms their exceptional stability and accuracy as normalizers. Therefore, the combination of *RlaEF1-α* and *RlaACT* is recommended as a robust internal control for reliable gene expression analysis in *Rhododendron* petal tissues.

## 4. Discussion

The accurate normalization of qRT-PCR data is fundamental to reliable gene expression studies, yet the selection of stable reference genes in non-model plants remains a significant challenge, particularly in genetically complex and phenotypically diverse genera such as *Rhododendron* [23]. This study presents the first systematic identification and validation of reference genes specifically tailored for expression analysis across Rhododendron cultivars with varying flower colors. Given the polyploid nature and extensive gene family expansions characteristic of many Rhododendron cultivars [24], the assumption that commonly used “housekeeping” genes from model plants will perform stably is untenable. Our comprehensive evaluation of 11 candidate genes across six distinct cultivars using four complementary algorithms (ΔCt, BestKeeper, NormFinder, and geNorm) was designed to address this methodological gap. The consistent identification of *RlaEF1-α* and *RlaACT* as the most stable genes—and the clear instability of *RlaPP2A* and *RlaHIS*—empirically underscores the necessity of context-specific validation. By establishing a robust, dual-gene normalization framework (*RlaEF1-α* + *RlaACT*) and validating it through the biologically coherent expression pattern of the anthocyanin-related gene *RlaMYB113*, this work provides an essential molecular toolkit for precise gene expression analysis in *Rhododendron*.

Our findings contribute to the growing body of literature on reference gene stability in horticultural plants and highlight both conserved and context-dependent patterns. While *ACT* is frequently employed as a reference across plant species, its stability is not universal [25,26]. Its strong performance here aligns with studies in several ornamentals but contrasts with reports in others where it varied significantly, reinforcing the need for empirical verification within each experimental system. Similarly, the top-ranking stability of *RlaEF1-α* is consistent with emerging evidence from other ornamental genera such as *Chrysanthemum* and *Rosa* [27], where elongation factors often outperform traditional references like *GAPDH* or *UBQ* [28,29]. The exceptional stability of *RlaEF1-α* and *RlaACT* can be attributed to their core housekeeping functions: *RlaEF1-α* is central to translation elongation, a process tightly regulated to maintain cellular homeostasis; *RlaACT* encodes a cytoskeletal component with typically constitutive expression, less responsive to metabolic reprogramming such as anthocyanin accumulation. Both are evolutionarily conserved and buffered against pathway-specific fluctuations, making them reliable internal controls for flower color studies. Conversely, the marked instability of *RlaPP2A* and *RlaHIS* illustrates the pitfalls of adopting reference genes without validation. Notably, these genes also exhibited suboptimal amplification efficiencies (116.61% and 118.27%, respectively) and, in the case of *RlaHIS*, a slightly lower R^2^ (0.9738), which may have contributed to their poor stability rankings. These genes, involved in signal transduction and chromatin organization, likely exhibit expression plasticity in response to developmental or physiological cues linked to flower coloration—a responsiveness that renders them unsuitable as stable normalizers in this context. This divergence from their stable performance in some annual plants underscores that reference gene suitability is intimately tied to biological system and experimental conditions, further emphasizing the imperative of systematic screening.

Beyond methodological contributions, this study has significant implications for Rhododendron functional genomics and molecular breeding. The validated reference genes will enable accurate expression profiling of key regulatory and biosynthetic genes within the flavonoid pathway, accelerating the discovery of genetic determinants underlying flower color variation. Furthermore, the integrative analytical framework established here—combining multi-algorithm stability assessment with experimental validation using a trait-relevant target gene—provides a replicable model for reference gene selection in other understudied perennial ornamentals. While the current study focused on petal tissues at a specific developmental stage, future investigations should evaluate the stability of *RlaEF1-α* and *RlaACT* across diverse tissues, developmental phases, and abiotic stress conditions to fully assess their broad utility within the genus. Nevertheless, for studies targeting pigment accumulation in Rhododendron flowers, the *RlaEF1-α* + *RlaACT* combination is unequivocally recommended as a robust internal control. By providing this foundational and validated toolset, our work removes a key technical barrier and paves the way for high-confidence gene expression studies, ultimately supporting the advancement of molecular breeding strategies aimed at enhancing ornamental traits in this ecologically and economically significant genus.

## 5. Conclusions

In summary, this study systematically evaluated the expression stability of 11 candidate reference genes across six flower-color cultivars of *R. lapponicum* using four independent algorithms (ΔCt, BestKeeper, NormFinder, and geNorm) and the RefFinder comprehensive ranking. Under the experimental conditions tested (petal tissues at full-bloom stage), *RlaEF1-α* and *RlaACT* were consistently identified as the most stable reference genes (geNorm M = 0.42 and 0.45; V2/3 = 0.112 < 0.15), while *RlaPP2A* and *RlaHIS* were the least stable. Validation with the anthocyanin-related gene *RlaMYB113* showed that only the optimal pair (*RlaEF1-α* + *RlaACT*) produced a biologically coherent expression pattern (>8-fold higher in red-flowered vs. white-flowered cultivars), supporting their reliability for this specific experimental context. We therefore recommend this dual-gene combination as a reliable normalization framework for future qRT-PCR studies on flower color development in *R. lapponicum*.

## Figures and Tables

**Table 1:** Primer sequences and amplification efficiency of 14 candidate reference genes and 1 target gene in *R. lapponicum*.

Gene	Gene Description	Primer Sequence F/R (5′–3′)	Product Size (bp)	Efficiency (%)	R^2^
*Rlaα-TUB1*	Alpha-tubulin1	GACCCACGCCACGGCAAGTA	172	99.91	0.9940
		CAACAGTCGGCGGCTGGTAG			
*Rlaα-TUB2*	Alpha-tubulin2	GCCACTATCAAGACCAAGCG	180	99.88	0.9902
		ATCAATGCGGGAGAACACCT			
*RlaDnaJ*	Chaperone protein DnaJ 49	TTGAGGATGTTCGGAAAGCA	204	107.58	0.9951
		ACCCATTAAACCCTTGAGCC			
*RlaCDC2*	Cyclin Dependent Kinase-putative	TCCCGTCACTATTCCACTCC	158	98.11	0.9937
		CCAGGCCATGTATCCTCATT			
*RlaACT*	Actin	AGCACCCAGTTCTTCTTACA	213	97.43	0.9922
		GGGCATAACCCTCATAGATAG			
*RlaH2A*	Histone H2A	CACGTCGAGGAGCAGCAAAG	162	100.66	0.9953
		TTCCCAGCCAACTCAAGCAC			
*Rla* *PP2A*	Protein phosphatase 2A	GCCGAAGAGGAAGAACAGAC	152	116.61	0.9933
		AAGTCAAGCAATCGCAACAC			
*Rla* *HIS*	Histone superfamily protein H3	AAGGCTGCTCGTAAGTCTGC	136	118.27	0.9738
		GGAATGGGAGTTTCCTGATA			
*Rla* *EF1-α*	Elongation factor 1-alpha	ATGATTCCGACCAAGCCTAT	126	100.21	0.9918
		ATCCTTCTTCTCCACGCTCT			
*Rla* *VHAC*	V-type proton ATPase subunit C	GGCGTGGTCAGTAGTTCGTT	222	100.70	0.9922
		ACCCTGCTTTCTGTTTATGG			
*Rla* *OTU*	OTU-like cysteine protease family protein	GGGACCCAGTTCTTCTACTTC	173	104.38	0.9922
		CATCACTTCTAGCCGCCTTA			
Target gene					
*Rla* *MYB113*	MYB transcription factors	TGTCTTCTGAGGAAGTGCGTTGA	211	100.37	0.9921
		GAGCGAGTGAGTGCCTGTTGC			

**Table 2:** Analysis results of Delta Ct, BestKeeper, and NormFinder software.

Rank	Delta Ct		BestKeeper			NormFinder	
	Gene	SDΔCt	Gene	SD	CV	Gene	S
1	*ACT*	0.59	*EF1-α*	0.64	2.71	*ACT*	0.109
2	*EF1-α*	0.63	*ACT*	0.72	2.94	*EF1-α*	0.117
3	*α-TUB1*	0.72	*CDC2*	0.78	3.09	*α-TUB1*	0.207
4	*OTU*	0.74	*DnaJ*	0.85	3.37	*CDC2*	0.214
5	*VHAC*	0.88	*α-TUB1*	0.93	3.54	*DnaJ*	0.306
6	*DnaJ*	0.92	*H2A*	0.98	3.68	*VHAC*	0.364
7	*CDC2*	0.97	*α-TUB2*	1.17	4.19	*α-TUB2*	0.406
8	*α-TUB2*	1.18	*VHAC*	1.25	4.54	*H2A*	0.669
9	*H2A*	1.24	*OTU*	1.41	4.61	*OTU*	0.718
10	*PP2A*	1.41	*PP2A*	1.78	5.16	*PP2A*	0.817
11	*HIS*	1.55	*HIS*	2.11	6.85	*HIS*	0.982

**Table 3:** Stability rankings for 11 candidate reference genes evaluated by ΔCt, BestKeeper, NormFinder, geNorm, and the comprehensive RefFinder analysis.

Rank	Delta Ct	BestKeeper	geNorm	NormFinder	RefFinder
1	*ACT*	*EF1-α*	*EF1-α/ACT*	*ACT*	*ACT*
2	*EF1-α*	*ACT*		*EF1-α*	*EF1-α*
3	*α-TUB1*	*CDC2*	*CDC2*	*α-TUB1*	*α-TUB1*
4	*OTU*	*DnaJ*	*H2A*	*CDC2*	*CDC2*
5	*VHAC*	*α-TUB1*	*DnaJ*	*DnaJ*	*DnaJ*
6	*DnaJ*	*H2A*	*α-TUB1*	*VHAC*	*VHAC*
7	*CDC2*	*α-TUB2*	*α-TUB2*	*α-TUB2*	*α-TUB2*
8	*α-TUB2*	*VHAC*	*VHAC*	*H2A*	*OTU*
9	*H2A*	*OTU*	*OTU*	*OTU*	*H2A*
10	*PP2A*	*PP2A*	*PP2A*	*PP2A*	*PP2A*
11	*HIS*	*HIS*	*HIS*	*HIS*	*HIS*

## Data Availability

The original contributions presented in this study are included in the article and Supplementary Material. Further inquiries can be directed to the corresponding author.

## Supplementary Materials

The following supporting information can be downloaded at: https://www.mdpi.com/article/10.3390/cimb48050444/s1.
