# Supplementary material for: Selection and Validation of Stable Reference Genes for Accurate qRT-PCR Analysis of Flower Color Development in Rhododendron lapponicum
Source: Curr Issues Mol Biol. 2026 Apr 24;48(5):444. doi: 10.3390/cimb48050444 (PMC13204480; doi:10.3390/cimb48050444)
Supplement: Supplementary file 1 [file cimb-48-00444-s001.zip › Supplementary Figures.pdf]

Supplementary Materials

Selection and Validation of Stable Reference Genes for Accurate qRT-PCR Analysis of Flower Color Development in *Rhododendron lapponicum*

Liang Xu [1,†], Gang Lu [2,†], Fangwei Zhou [1], Congguang Shi [1], Xiaomei Zhu [3] and Shaozong Yang [1,*]

1 Zhejiang Key Laboratory of Forest Genetics and Breeding, Zhejiang Academy of Forestry, Hangzhou 310023, China; xuliang@zjforestry.ac.cn (L.X.); zhoufangwei@njfu.edu.cn (F.Z.); shicongguang@zjforestry.ac.cn (C.S.)
2 Zhejiang Forestry—Fund Management Center, Hangzhou 310012, China; 13738037801@163.com
3 Lin'an District Agriculture and Rural Affairs Bureau, Hangzhou 311300, China; zxm15906641535@163.com
* Correspondence: yangsz863@163.com
† These authors contributed equally to this work.

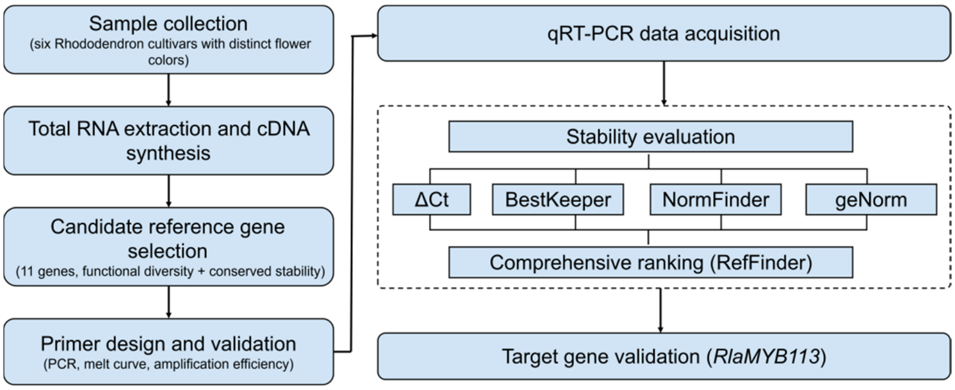

Figure S1. Schematic flowchart of the overall analytical pipeline for reference gene selection and validation in *R. lapponicum*.

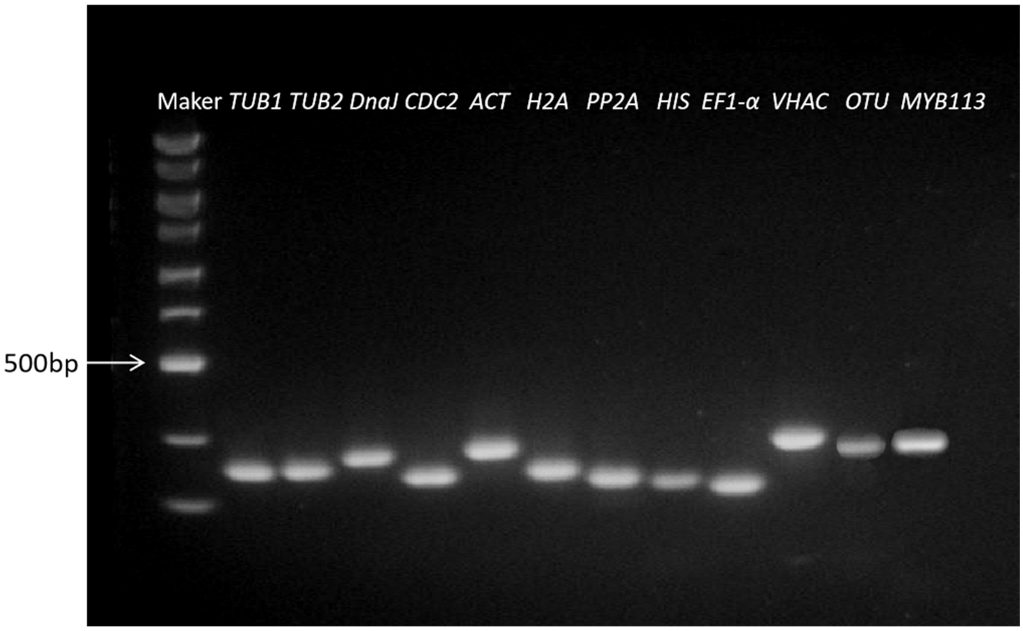

Figure S2. 1% agarose gel electrophoresis shows specific bands of PCR products.

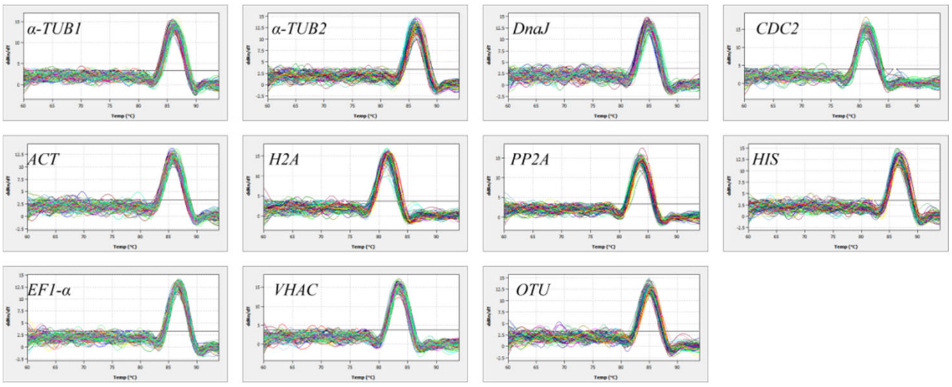

Figure S3. Melting curves of 11 reference genes showing single peaks.

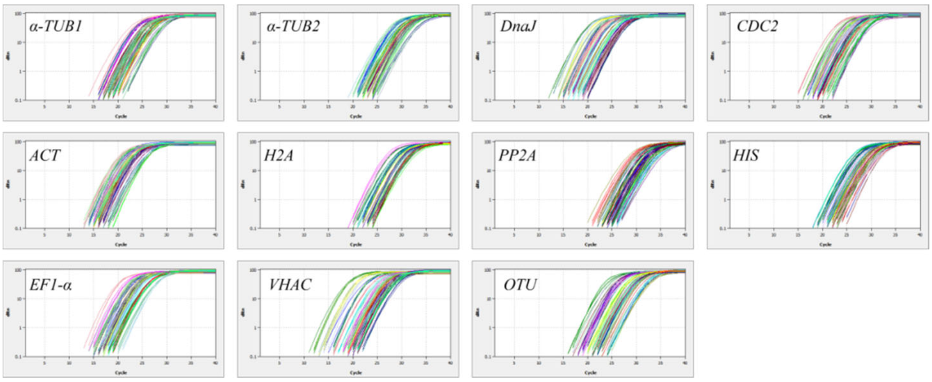

Figure S4. Amplification plots of the 11 candidate reference genes.
